# Dietary Curcumin Intake and Its Effects on the Transcriptome and Metabolome of *Drosophila melanogaster*

**DOI:** 10.3390/ijms25126559

**Published:** 2024-06-14

**Authors:** Samantha Belcher, Gerardo Flores-Iga, Purushothaman Natarajan, Garrett Crummett, Alicia Talavera-Caro, Celeste Gracia-Rodriguez, Carlos Lopez-Ortiz, Amartya Das, Donald A. Adjeroh, Padma Nimmakayala, Nagamani Balagurusamy, Umesh K. Reddy

**Affiliations:** 1 Department of Biology, Gus R. Douglass Institute, West Virginia State University, Institute, WV 25112, USA; sbelcher7@wvstateu.edu (S.B.); juan.iga@wvstateu.edu (G.F.-I.); pnatarajan@wvstateu.edu (P.N.); gcrummett@wvstateu.edu (G.C.); atalaveracaro@wvstateu.edu (A.T.-C.); celeste.rodreguez@wvstateu.edu (C.G.-R.); carlos.ortiz@wvstateu.edu (C.L.-O.); adas@wvstateu.edu (A.D.); padma@wvstateu.edu (P.N.); 2 Laboratorio de Biorremediación, Facultad de Ciencias Biológicas, Universidad Autónoma de Coahuila, Torreón 27275, Coahuila, Mexico; 3 Lane Department of Computer Science and Electrical Engineering, West Virginia University, Morgantown, WV 26506, USA; donald.adjeroh@mail.wvu.edu

**Keywords:** curcumin, phytochemicals, *Drosophila melanogaster*, RNA-seq, metabolomics

## Abstract

Curcumin, a polyphenol derived from *Curcuma longa*, used as a dietary spice, has garnered attention for its therapeutic potential, including antioxidant, anti-inflammatory, and antimicrobial properties. Despite its known benefits, the precise mechanisms underlying curcumin’s effects on consumers remain unclear. To address this gap, we employed the genetic model *Drosophila melanogaster* and leveraged two omics tools—transcriptomics and metabolomics. Our investigation revealed alterations in 1043 genes and 73 metabolites upon supplementing curcumin into the diet. Notably, we observed genetic modulation in pathways related to antioxidants, carbohydrates, and lipids, as well as genes associated with gustatory perception and reproductive processes. Metabolites implicated in carbohydrate metabolism, amino acid biosynthesis, and biomarkers linked to the prevention of neurodegenerative diseases such as schizophrenia, Alzheimer’s, and aging were also identified. The study highlighted a strong correlation between the curcumin diet, antioxidant mechanisms, and amino acid metabolism. Conversely, a lower correlation was observed between carbohydrate metabolism and cholesterol biosynthesis. This research highlights the impact of curcumin on the diet, influencing perception, fertility, and molecular wellness. Furthermore, it directs future studies toward a more focused exploration of the specific effects of curcumin consumption.

## 1. Introduction

Curcumin, the major isolated curcuminoid derived from the underground stem of *Curcuma longa* (turmeric), a member of the ginger family (Zingiberaceae) [1], has received considerable attention in recent years for its wide range of pharmacological effects such as antioxidant, anti-inflammatory, antimicrobial, antitumor, antiallergic, hepatoprotective, and neuroprotective activities [2,3,4,5,6]. It has been used for centuries as a spice in Asian food [7]. It is used worldwide as a dietary spice and in cosmetic and pharmaceutical industries [8].

Curcumin is a potent antioxidant due to conjugated double bonds in its chemical structure. It is an effective electron donor to counteract the production of reactive oxygen species (ROS) in several redox reactions [9,10]. Moreover, curcumin has been extensively used in the treatment of different medical conditions like cystic fibrosis, diabetes, gastric ulcers, liver diseases, arthritis, and osteopenia [11,12,13]. It has been reported that curcumin, as a dietary polyphenol, counteracts the effects of toxic damage in different tissues [14] and, in addition, it can interfere with key cancer-associated signaling pathways by directly targeting proteins or by the regulation of anti-apoptotic genes [15]. Curcumin is also reported as a preventive measure for treating Alzheimer’s disease [16,17]. Several other studies have also demonstrated that curcumin has strong neuroprotective antioxidant properties, for instance, scavenging ROS [18], and neutralizing nitric oxide-based free radicals [19]. However, adverse and negative side effects have also been reported related to dizziness, gastroesophageal reflux, an increase in alkaline phosphatase serum, lactate dehydrogenase levels, and a negative impact on fertility [20].

There is a great interest in how bioactive phytochemicals such as curcumin can be employed for medicinal purposes, leading to the study of their mechanisms and effects at the molecular level [21]. *Drosophila melanogaster*, the fruit fly, is an excellent biomodel for understanding different diets’ effects due to its short life span and handy manipulation [22,23]. Specifically, the genome similarity and presence of highly conserved metabolic pathways with mammals, e.g., humans, make the fruit fly an exceptional model for understanding the genetic and metabolic background of various diets and environmental interactions [24,25]. Curcumin studies in fruit flies have shown that curcumin attenuates oxidative stress by improving antioxidant activity [26], prolonging lifespan [27], and enhancing the immune response to neuropathologies such as Huntington’s disease [28,29]. Nevertheless, deterrent effects have been also shown in response to curcumin [30,31], specifically, decreasing the lifespan, larval development, and egg laying. Hormesis is a process in which the consumption of a substance, e.g., curcumin, can have priming and inhibitory effects in a dose-dependent manner [32,33], an effect that has been previously discussed in curcumin [34], and can be a reason for these discrepant results, as well as divergent physiological and genomic structure characteristics between vertebrates and invertebrates [35].

In the present study, we comprehensively analyze curcumin-mediated modulation of gene expression and metabolite concentration in *D. melanogaster* model in a fly-safe dosage [36], also in the range of human supplementation [37], by integrating transcriptome and metabolome analysis to explore the molecular basis of curcumin consumption and its effects on human health.

## 2. Results

### 2.1. Physiological Changes of Drosophila Melanogaster Fed with Curcumin

**Figure 1:**
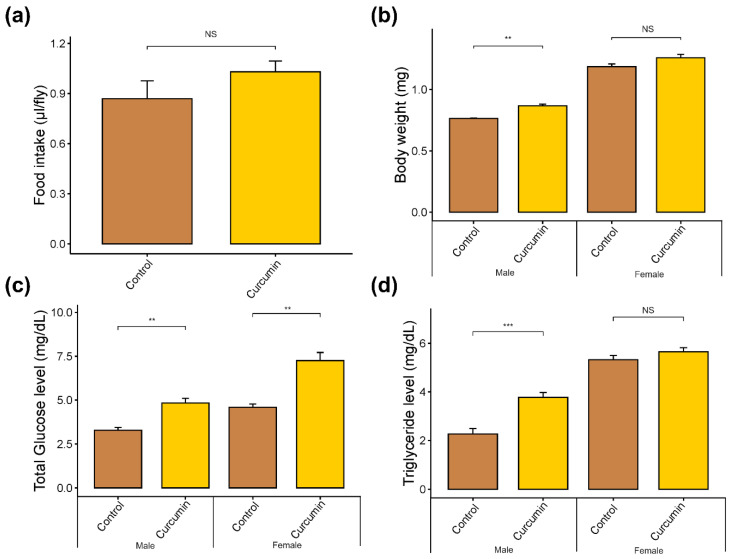
*D. melanogaster* food intake and physiological changes reared on control and curcumin diets. (**a**) CAFE assay in (µL/fly), (**b**) body weight (mg), (**c**) triglycerides content (mg·dL^−1^), and (**d**) glucose level (mg·dL^−1^) under control and curcumin diets. Data are mean ± SEM for n = 5 independent replicates. ** *p* ≤ 0.01, *** *p* ≤ 0.001, NS, not significant, compared to control versus treatment.

To determine the food consumption of flies in control and curcumin-supplemented diets, we used the capillary feeder assay (CAFE). We observed a slight increase of 0.16 µL/fly in food consumption when males and females were fed on the curcumin-supplemented diet compared to the control (Figure 1a). Still, this difference was not statistically significant (*p*-value > 0.05, Cohen’s d = 0.91). The total food consumption was 0.86 ± 0.21 µL/fly and 1.03 ± 0.12 µL/fly for the control and curcumin diets, respectively. To check if there was a sex bias in our results, we assessed the food consumption for males and females separately (Figure S1). Similarly, no statistically significant changes for food consumption were detected.

Body weight, triglyceride, and glucose levels were determined on 5-day-old mature flies reared on either curcumin or a control diet to obtain insights into the physiological changes when flies are fed with curcumin. For both sexes, the body weight slightly increased in the curcumin diet (Cohen’s d < 0.2). However, this increase was only statistically significant in male flies (*p* = 0.01), with a mean difference of 0.10 mg compared to the control (Figure 1b). Moreover, we measured triglyceride and total glucose levels to assess the implications of increased body weight. Triglyceride content was not statistically significant in female flies (*p* > 0.05), but a high glucose increment of 2.36 mg/dL was observed when fed with the curcumin-supplemented diet (*p* < 0.01, Cohen’s d > 0.7) (Figure 1c,d). In addition, male flies exhibited an increase of 1.5 mg/dL for glucose and triglycerides compared to the control group (Figure 1c,d).

### 2.2. RNA-Seq Data of Flies in a Curcumin Diet

**Figure 2:**
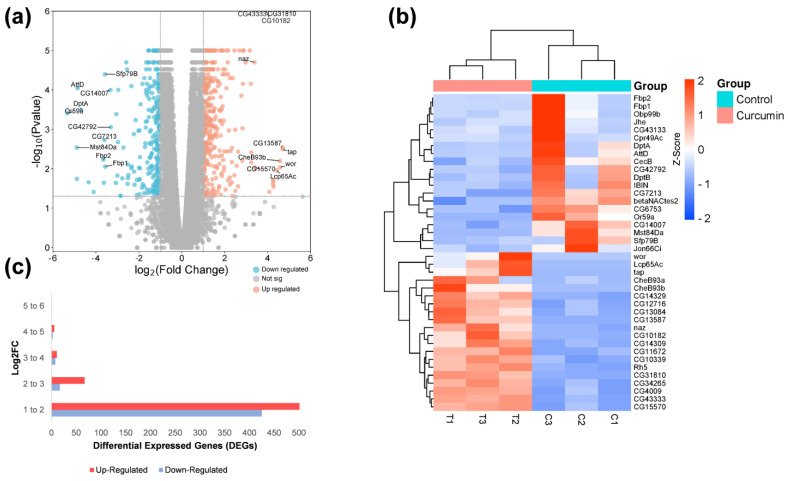
Gene expression changes between *D. melanogaster* reared on curcumin versus control diet. (**a**) Volcano plot of RNA-seq with a fold change threshold of >1.0 (*x*-axis) and *p*-value threshold < 0.05 (*y*-axis). Blue and red circles represent up-and down-regulated genes above the threshold. (**b**) Heatmap of the top and bottom 20 genes with the highest expression variability. The color key represents normalized count values. The top dendrogram shows the tissue and side dendrogram relationships among genes. (**c**) Fold change distribution of DEGs of Drosophila fed with curcumin vs. control diet.

To obtain a comprehensive overview of the impact of curcumin on gene expression in *D. melanogaster*, we analyzed RNA-seq data obtained from flies reared on control and curcumin diets. The cDNA libraries were sequenced using an Illumina NextSeq500 platform, producing 184,079,746 raw reads. After quality control filtering (Q > 30), 14.44% of the reads were removed, leaving 157,504,046 clean reads (Table S1). The reads were subsequently mapped to the *D. melanogaster* reference genome, with 96.6 to 97.5% of the reads being successfully mapped. A principal component analysis (PCA) revealed that the gene expression under control and curcumin diets were clustered according to the biological replicate and separated in the PC1 × PC2 score plot according to the diet differentiation by PC1 (62%) and PC2 (21%) (Figure S2). In our differentially expressed genes (DEG) analysis, we identified 1043 differentially expressed genes (DEGs) with a fold change of ≥1, comparing the curcumin to the control diet (Table S2). Of these, 589 were upregulated, and 453 were down-regulated. The top up-and down-regulated genes, logFC distribution, and a heatmap of the top 40 (20 up- and 20 down-regulated genes) are shown in Figure 2a–c.

### 2.3. Gene Ontology, Networks, and Pathways Found in DEGs

**Figure 3:**
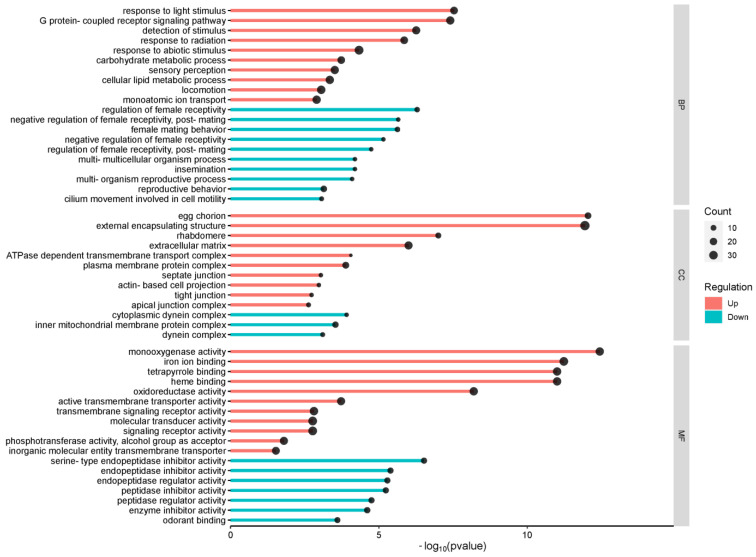
Top biological process (BP), cellular component (CC), and molecular function (MF) found in up- and down-regulated genes between *D. melanogaster* reared on curcumin versus control diet.

The identified DEGs were annotated using the BLASTx algorithm with the FlyBase database. Gene ontology (GO) enrichment was performed for biological processes, cellular components, and molecular functions using TopGO, resulting in 185 terms (Table S3). The top GO terms in the DEGs (Figure 3) were “response to abiotic stimulus,” “locomotion cellular,” “lipid metabolic process,” “monoatomic ion transport,” and “G protein-coupled receptor signaling pathway” for biological processes. For cellular components, the top GO terms were “external encapsulating structure,” “extracellular matrix,” “plasma membrane protein complex,” “egg chorion,” and “inner mitochondrial membrane protein complex”. The most abundant GO terms for molecular function were “signaling receptor activity”, “molecular transducer activity”, “iron ion binding”, “transmembrane signaling receptor activity”, and “monooxygenase activity”.

**Figure 4:**
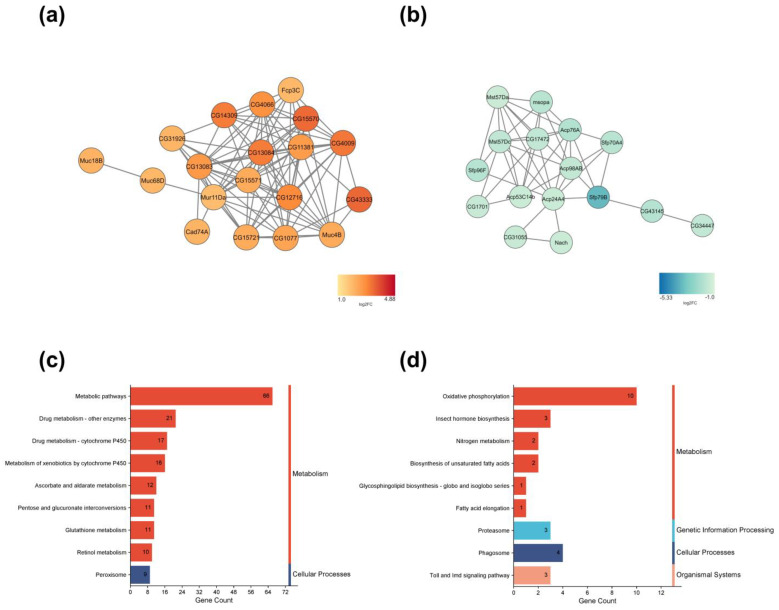
Largest gene linkage subnetworks and pathways of up-and down-regulated DEGs in response to a curcumin diet. Largest subnetwork of (**a**) upregulated and (**b**) down-regulated genes, cutoff of *p*-value < 0.05. Below, color scales indicate the log2FC of each displayed gene. Top 10 KEGG pathways count in (**c**) upregulated and (**d**) down-regulated genes.

To examine the relationship between terms and functional groups in biological networks, we utilized ClueGO in Cytoscape. We queried the top 100 up-and down-regulated genes (Figure S3) and then filtered to the largest network of genes (Figure 4 and Table S4). For the upregulated genes, we found that the entire largest network of genes was associated with “external encapsulating structure organization,” “cell periphery,” and “extracellular matrix structural constituent” (Figure 4a). On the other hand, for the down-regulated genes, we found that “sexual reproduction,” “extracellular region,” and “serine-type endopeptidase inhibitor activity” were found (Figure 4b).

We used the Kyoto Encyclopedia of Genes and Genomes (KEGG) pathway database to analyze the pathways of DEGs. One hundred thirty pathways were assigned to the DEGs, with 85 upregulated and 35 down-regulated pathways (Table S5). The top ten gene count pathways were classified into KEGG categories. The pathways “Metabolism” and “Cellular Processes” were common for both up-and down-regulated pathways, while “Genetic Information Processing” and “Organismal Systems” were unique for down-regulated pathways. The upregulated DEGs were found to have more genes in “Metabolic Pathways,” “Drug Metabolism—Other Enzymes,” “Drug Metabolism—Cytochrome P450”, “Metabolism of Xenobiotics by Cytochrome P450”, and “Ascorbate and Aldarate Metabolism,” (Figure 4c). Down-regulated DEGs had more genes in “Oxidative Phosphorylation,” “Phagosome,” “Proteasome,” “Insect Hormone Biosynthesis,” and “Toll and IMD Signaling Pathway” (Figure 4d).

### 2.4. DEGs in Response to CURCUMIN

Among the top 10 up and down DEGs in response to curcumin ingestion, we found genes related to “oxidoreductase activity” (*CG31810*), “response to sugar and salt” (*tap*), “pheromone detection chemosensory proteins” (*CheB93b*) and “Involved in sperm assembly” (*Mst84Da*), “Involved in sexual reproduction” (*Sfp79B*), and “immunity inducible peptide having activity against gram-negative bacteria” (*AttD* and *DptA*) (Table S2).

Moreover, we focused on investigating genes related to gustatory perception, as curcumin is widely used in food as a dietary spice. A total of 15 related genes, odorant-binding (11), odorant-receptor (3), and gustatory receptor (1) genes, were found among the top up-and down-regulated DEGs towards the perception of curcumin supplemented in the diet (Table S6) being an odorant receptor, *Or59a* the most down-regulated gene in all the DEGs. Moreover, we observed the up-regulation of three chemosensory proteins, CheB93a, CheA75a, and CheA87a, related to pheromone detection, defense against fungal infection, and chemical stimulus, respectively.

Additionally, to comprehend the physiological consequences of curcumin in flies, we investigated genes related to oxidative stress, fat metabolism, and sugar metabolism in the DEGs, as shown in Table 1. We identified three upregulated genes associated with the oxidative stress relief process: *CG4009*, *Cyp6d2*, and *CG2065*. In terms of fat metabolism, we observed the up-regulation of *Fad2*. This transmembrane fatty acid desaturase utilizes mono-unsaturated long-chain fatty acids (LCFAs) to synthesize di-unsaturated LCFAs, Lsd-1, a protein associated with protecting lipid droplets from lipase-mediated remobilization and regulating lipid storage amounts, Traf-like, related to the positive regulation of lipophagy, *CG1946*, involved in the triglyceride biosynthetic process, *FASN1*, involved in glycogen metabolism and triglyceride biosynthesis, *CG17562*, involved in the long-chain fatty-acyl-CoA metabolic process, *CG8306*, which catalyzes the reduction of C_16_ or C_18_ fatty acyl-CoA to fatty alcohols, and the down-regulation of three triacylglycerol lipases *CG18530*, *CG11608*, and *CG6753*. Concerning sugars, we identified the up-regulation of *dawdl*, which regulates insulin in neurons, *tequila*, involved in glucose homeostasis, memory, and the positive regulation of insulin secretion, *CG4797*, a glucose-trehalose transmembrane transporter. Additionally, there was an up-regulation of *Akh*, an adipokinetic hormone secreted by the corpora cardiac involved in energy processes such as hemophilic carbohydrate circulation and the storage of lipids and glycogen.

### 2.5. Metabolic Changes in Drosophila under Curcumin Treatment

**Figure 5:**
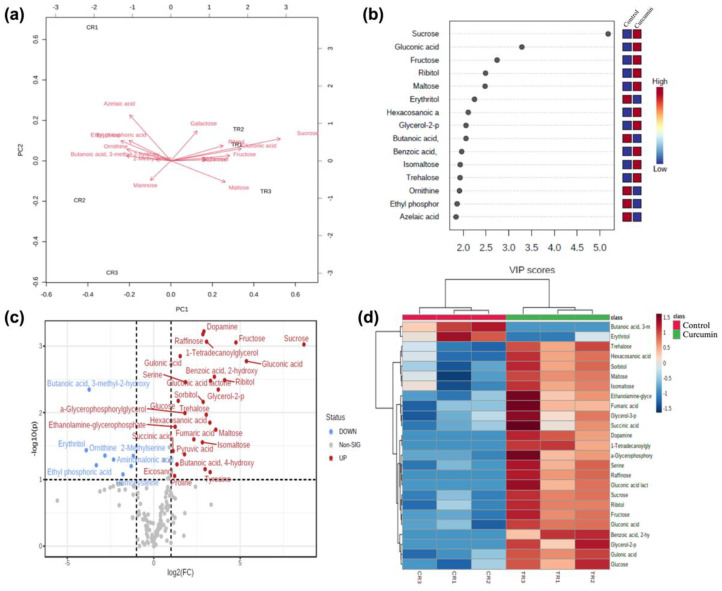
Untargeted metabolomics of *D. melanogaster* reared on curcumin diet. (**a**) Principal component analysis (PCA) biplot between the top 15 selected metabolites in the control and treatment diet. (**b**) Important compounds identified by PLS-DA in Drosophila. The colored boxes on the right indicate the relative concentration of the corresponding metabolite. (**c**) Volcano plot visualization of the important metabolites selected by both fold change and *p*-value. The red circles represent the upregulated metabolites, while the blue circles represent the down-regulated metabolites. (**d**) The top 25 most important metabolites by area response of *D. melanogaster* in the two different diet treatments: control (CR) and curcumin (TR).

To investigate the main metabolic changes reflecting the variation in diet, we conducted GC/MS metabolite profiling and multivariate statistical analysis to determine the changes in the *D. melanogaster* metabolome induced by curcumin (Table S7). The PCA analysis revealed a clear separation between the curcumin-treated and control groups, with the principal components being PC1 (49.6%) and PC2 (20.6%) (Figure S4). After analyzing the top 15 metabolites using PCA, we found that they represented PC1 (80.9%) and PC2 (5%) of the total variations (Figure 5a). Galactose, ribitol, gluconic acid, sucrose, raffinose, and dopamine positively contributed positively to the flies under the curcumin diet, while maltose showed a negative contribution. Moreover, partial least square discrimination was employed for the classification of differentially accumulated metabolites (DAMs), with sucrose, gluconic acid, fructose, ribitol, maltose, dopamine, glycerol-2-p, 2-hydroxy benzoic, and trehalose showing highly different concentrations in response to curcumin diet when compared to the control (Figure 5b).

Log2FC and *p*-value calculations identified 73 DAMs (Table S8). Among them, 41 showed an increased concentration, while 32 exhibited a decrease. The distribution of these metabolites is illustrated in a volcano plot (Figure 5c), emphasizing dopamine, raffinose, sucrose, and fructose as the most significantly upregulated compounds and butanoic acid, erythritol, and ornithine as the most prominently down-regulated. An analysis of the top 25 DAMs in Drosophila using a heatmap (Figure 5d) unveiled a significant influence of curcumin supplementation on specific metabolite production. Remarkably, the group receiving curcumin showed elevated levels of essential metabolites like sucrose, gluconic acid, fructose, ribitol, glycerol, maltose, benzoic acid, galactose, tyrosine, dopamine, sorbitol, and raffinose compared to the control group. Conversely, azelaic acid, erythritol, butanoic acid, ethyl phosphoric acid, hexadecanol, galactitol, ornithine, tridecanoic acid, mannose, and pentanoic acid were found to be down-regulated.

### 2.6. Enrichment Analysis of DAMs

**Figure 6:**
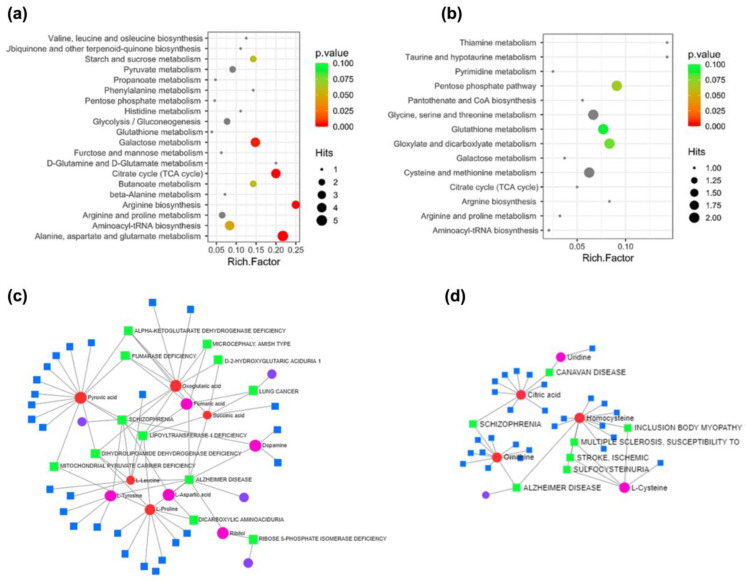
KEGG pathways enrichment comparison of (**a**) up- and (**b**) down-regulated metabolites under curcumin versus control diet. Metabolite–disease interaction network analysis of the top (**c**) up- and (**d**) down-regulated metabolites in *D. melanogaster* under a curcumin-supplemented diet. Enriched terms are represented as nodes, and the node size represents the significance of each term. Red and pink circles represent high and low metabolite-disease correlation, respectively. Green squares represent the associated disease.

To enhance our understanding of the biological functions of various metabolites in response to curcumin treatment, we conducted KEGG functional annotation and pathway enrichment analysis of DAMs. In the case of up-accumulated metabolites, we identified 20 KEGG pathways associated with key metabolic processes (Figure 6a). These pathways include galactose metabolism, citrate cycle (TCA cycle), arginine biosynthesis, and alanine, aspartate, and glutamate metabolism. Conversely, 14 KEGG pathways were identified for down-accumulated metabolites, encompassing glycine, serine, and threonine metabolism and cysteine and methionine metabolism (Figure 6b).

Additionally, we constructed a metabolite–disease interaction network using up- and down-DAMs (Figure 6c,d). From the up-DAMs, we identified associations between pyruvic acid, L-leucine, and oxoglutaric acid with diseases such as schizophrenia, lipoyltransferase 1 deficiency, and dihydrolipoamide dehydrogenase deficiency. Succinic and fumaric acids were linked to lung cancer and lipoyltransferase 1 deficiency. L-proline correlated with dicarboxylic aminoaciduria, while ribitol was associated with ribose 5-phosphate isomerase deficiency. Oxogluratic acid was found to be linked to diseases such as fumarase deficiency, alpha-ketoglutarate dehydrogenase deficiency, microcephaly—Amish type, and d-2-hydroxyglutaric aciduria 1. We also found L-proline, L-leucine, succinic acid, dopamine, L-aspartic acid, and L-tyrosine associated with Alzheimer’s disease. Moreover, within the network of down-DAMs, citric acid was identified, showcasing connections to Canavan disease and schizophrenia. Ornithine was implicated in schizophrenia and Alzheimer’s disease, while homocysteine exerted influences on Alzheimer’s disease, encompassing body myopathy, multiple sclerosis, stroke, and sulfocysteinuria.

### 2.7. Weighted Gene Co-Expression Network Analysis (WGCNA)

**Figure 7:**
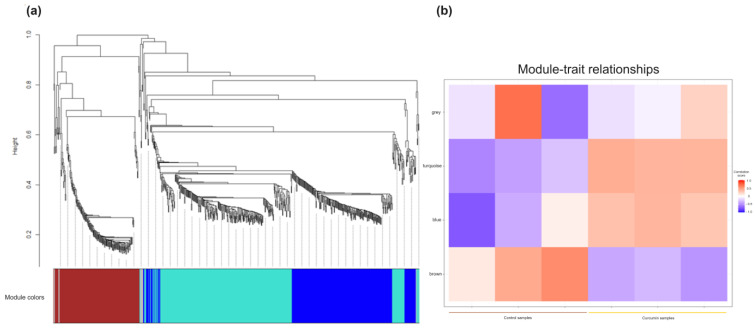
WGCNA analysis results. (**a**) A hierarchical cluster dendogram showing co-expression modules identified and (**b**) heat map analysis of the different modules in control and curcumin diets.

To better understand the interplay between genes and metabolites, we first performed a WGCNA analysis (Figure 7) involving clustering DEGs into four modules according to their correlation. Among these modules, two exhibited the highest correlation values. The turquoise module was associated with arginine-proline metabolism (*Gclc*, *Aldh7A1*, *Odc1*), G protein-coupled receptor (GPCR) downstream signaling (*Mmp1*), glycine-serine and threonine metabolism, dihydropyrimidine dehydrogenase deficiency (*Gnmt*, *Srr*), purine and nucleotide metabolism (*Gart*, *Pfas*), and DNA repair (*Rchy1*). On the other hand, the blue module was linked to alanine and aspartate metabolism (*Gad1*), protein digestion and absorption (*Col4a1*), glyoxylate and dicarboxylate metabolism [13], and signal transduction (*Col4a1*, *FER*, *Apc*, *SPARC*, *Mmp1*, *AGO2*). In contrast, the brown module exhibited low correlation values in response to curcumin treatment, particularly concerning the pentose phosphate pathway, glucagon signaling pathway, metabolism of carbohydrates, glucose, and gluconeogenesis. Meanwhile, the gray module comprised genes lacking numeric correspondence to our transcripts, and these genes were not clustered into any specific module.

### 2.8. Transcriptome and Metabolome Integration

To better understand the interplay between genes and metabolites, we integrate our transcriptome and metabolome analysis with IMPaLA [38]. This involved overlapping genes present in curcumin-correlated modules from our WGCNA, with DAMs. Table 2 highlights the top 10 common pathways between genes and metabolites, while a detailed compilation of shared genes and metabolites within each module can be found in Table S9. These pathways include arginine proline metabolism, alanine, and aspartate metabolism, glutamate glutamine metabolism, G protein-coupled receptors (GPCR) downstream signaling, protein digestion and absorption, glycine, serine, and threonine metabolism, gPcR signaling, glyoxylate and dicarboxylate metabolism, purine metabolism, and purine nucleoside phosphorylase deficiency. Each pathway plays a critical role in various biological processes. For example, arginine proline metabolism involves the biosynthesis of essential amino acids such as arginine, ornithine, proline, citrulline, and glutamate, while alanine and aspartate metabolism provide energy through gluconeogenesis and NH_3_ transport. Similarly, glutamate glutamine metabolism supports nucleotide synthesis and NH_3_ transport, and GPCR downstream signaling activates critical signaling pathways involving cAMP and inositol phospholipids. Protein digestion and absorption ensure the production of amino acids and small peptides, and glycine, serine, and threonine metabolism contributes essential amino acids and glycolysis intermediates.

This study also highlights the involvement of specific genes and metabolites in these pathways. Key genes include *GCLC*, *GAD1*, *ALDH7A1*, *MMP1*, *COL4A1*, *GNMT*, *SRR*, *CAT*, *GART*, and *PFAS*, and metabolites such as glycine, α-ketoglutaric acid, UREA, succinic acid, aspartic acid, GABA, histamine, threonine, pyruvic acid, dopamine, stearic acid, sucrose, hypoxanthine, xanthine, adenine, guanosine, and fumaric acid were identified, indicating the biochemical changes occurring in response to curcumin intake. These findings suggest that curcumin influences multiple metabolic and signaling pathways, potentially affecting oxidative stress response, amino acid metabolism, and energy production. Results from this study underscore the importance of further exploration of specific genes like *Nrf2* homologs and stress response mechanisms using specific techniques such as RT-qPCR and knockdown/knockout strains to understand curcumin’s impact fully. Additionally, the research highlights the relevance of studying curcumin’s effects across different dosages and forms, considering the broader implications for both invertebrates and humans.

## 3. Discussion

Curcumin is a polyphenolic compound and is the primary active agent of turmeric (*Curcuma longa* L.), a perennial herbaceous herb with the underground stem that accumulates curcumin commonly used as a food additive for flavor, preservation, and color [39]. This compound has been linked to various health benefits, such as its ability to fight cancer, its antioxidant and anti-inflammatory properties, and its potential to modulate the immune system [40]. Although there is a bias inclination toward only studying the positive effects of curcumin [41], curcumin’s chemical structure contains 2 a,b-unsaturated ketones, which have been associated with adverse health effects, including genotoxicity, thyroid gland follicular cell hyperplasia, and reduced fertility [42,43].

To investigate the physiological effects of curcumin consumption and their underlying molecular mechanisms, we used a transcriptomic and metabolomic approach in *D. melanogaster* to address this question, a model organism that shares over 70% of genetic homology with humans [44]. Fly research on curcumin supplementation has been associated with improving life span [27,45,46,47], relief of oxidative stress through restoring enzymatic and non-enzymatic antioxidant mechanisms [19,45,48,49,50,51], and as a neuroprotectant therapeutic agent for Alzheimer’s and Huntington’s neurodegenerative diseases [10,28,52,53]. In our transcriptomic analysis, we found a gene with oxidoreductase activity, *CG31810*, as the top upregulated gene, and up-regulation of arginine metabolism, related to promoting in adult flies an antioxidant response and increased lifespan, acting directly against oxidative stress [54]. Similarly, we identified the up-regulation of alanine, aspartate, and glutamate metabolic pathways, reported as an essential signal modulator associated with aging [55], derived from central metabolism (TCA). On the other hand, methionine, serine, and glutathione pathways were down-regulated in response to curcumin diet. Previous studies have reported that curcumin’s anti-carcinogenic, antioxidant, and cytoprotective [36,56] effects could be attributed to its inhibitory impact on glutathione S-transferase and glutathione synthesis against Parkinson’s disease [57,58]. Likewise, higher serine levels have been observed to cause worse cognitive function in Alzheimer’s disease [59], and methionine restriction decreased carcinogenic processes and prevention of neurodegenerative diseases [60,61]. In our results, we did not find the transcription up-regulation of enzymatic antioxidant mechanisms such as superoxide dismutase or catalase, which are linked to enhanced activity in the presence of curcumin in mammals [62,63]. This might be due to our form of curcumin supplementation that did not include the addition of bioavailability enhancers such as piperine, the dosage used in our study that can inhibit these antioxidant enzymes, and the limitations of translating these mechanisms to higher organisms [20,45]. Furthermore, we found that the arginine-proline pathway showed high correlation values between DEGs and DAMs; this pathway is one of the central pathways for the biosynthesis of arginine and proline from glutamate. Proline is a chaperone able to stabilize proteins in their natural conformation [64], suggesting that a curcumin diet may affect protein levels and stability. Moreover, we found dopamine, pyruvic acid, proline, leucine, and oxoglutaric acid in our DAMs. It has been shown that adding curcumin to the diet improves lifespan and significantly alters gene expression [46] with a correlation with these metabolites, with dopamine being the most important dopaminergic involved in maintaining the nervous system [65]. Taken together, we were able to screen that curcumin had a positive effect on neuroprotection in *D. melanogaster*, with a special prevention in diseases such as Alzheimer’s, schizophrenia, and multiple sclerosis, and counteracting effect of reactive oxygen species (ROS). Nonetheless, it is interesting to note that our results does not show the modulation of *cnc*, the homologous gene for *Nrf2* in the fruit fly [66], which is linked to the beneficial effects of curcumin such as its anti-aging, neuroprotective, antioxidant, and anticancer properties [67].

Interestingly, we observed a 19% increase in fly consumption with curcumin supplementation, suggesting that flies may consume more significant amounts of food when the diet contains curcumin [48]. Although a bodyweight increase has not been reported on other mammal models fed with curcumin [68], in a Huntington disease *Drosophila* strain [10], it was found that curcumin increased the weight of male flies. In addition, one of the significant DAMs in response to curcumin consumption was dopamine. It has been reported that higher dopamine levels are linked to an increased incentive salience or craving for food-related rewards, thus contributing to the initiation of food consumption [56,69]. Concerning gustatory and odor responses, *Or59a* was the most down-regulated gene. This gene is expressed in olfactory receptor neurons and is responsible for mediating the response to volatile chemicals with benzene rings, such as curcumin [70,71]. Moreover, the *tap* gene, related to sugar and salt response, which interacts with the Wnt-PCP pathway to regulate neuronal extension and guidance, was highly upregulated [72,73]. Whether the transcriptional inhibition of *Or59a* and the high up-regulation of the *tap* gene are explicitly associated with curcumin and drive behavioral, dietary preferences remains an area for further investigation. Furthermore, curcumin is linked to enhanced fatty acid oxidation and promotes carbohydrate homeostasis [74]. However, we noticed a significant high increment effect on the total glucose and triglycerides levels for both fly sexes subjected to the curcumin diet (Cohens’ d > 0.8). Likewise, we found three upregulated sugar metabolism-related genes, *dawdl*, *tequila*, and *CG4797*, and three triacylglycerol lipases being down-regulated: *CG18530*, *CG11608*, and *CG6753*. Accordingly, sucrose, fructose, and glycerol-3-phosphate metabolites were found to be up-accumulated and used as an energy cell source. Gluconic acid, dopamine, and raffinose were found to be highly accumulated. In contrast, erythritol was down-accumulated, associated with a shorter lifespan and the lowest fecundity in *Drosophila suzukii* [17,75]. Studies have shown that curcumin is metabolized mainly by reduction and conjugation with glucuronic acid and sulfate [76], producing a metabolome shift in response to curcuminoids [6]. Moreover, the pathway analysis based on enrichment reveals the up-regulation of intermediates involved in amino acid biosynthesis and tricarboxylic acid cycle (TCA) pathway, participating in the balanced energy production and suggesting that their activity is needed in the process to metabolize curcumin by NADPH-dependent enzymes [76]. These findings might explain the discriminatory preference for food with higher carbohydrate content and less protein, such as that seen in Asian cousins [77].

One of the main properties associated with curcumin is the enhancement of recovery [10,78]. We found three genes related to this process were upregulated: *Starvin, Hand,* and *p38c*, which are related to somatic muscle maintenance and stress response [79], vertebrate cardiogenesis and muscular development hemostasis [80], and stress and wound responses to activate anti-inflammatory cells [81]. Therefore, they contribute to the healing effects associated with curcumin.

Curcumin has been acknowledged for its impact on reproductive processes and is advised against during conception due to its known effects on fertility and egg development [43,82,83,84]. In *D. melanogaster*, curcumin has been shown to influence egg hatching and delay larval development [27]. Our analysis revealed that the most significantly down-regulated gene network included genes associated with “A type of reproduction that combines the genetic material of two gametes (such as a sperm or egg cell or fungal spores)”, “The specific behavior of a female organism that is associated with reproduction”, and “mating, insemination, and reproductive behavior” in the down-regulated biological process. Notably, *Sfp79B* was the most down-regulated gene in the network, coding for the seminal fluid protein, impacting male reproduction and influencing male sperm competition and female post-mating behavior [85,86]. Consistent with our gene expression data, elevated levels of arginine identified in our metabolome profile are associated with decreased fecundity [87]. Henceforth, our results support the assertion that curcumin impacts reproductive success. However, further research on developmental impact and reproductive behavior is needed to elucidate the sex-specific effects on reproduction and mating behavior, which will refine our understanding.

The polyphenolic structure of curcumin enables its function as an antibiotic through the deterioration of bacterial quorum sensing communication system, gene and protein expression, and integral cell structures of several strains of gram-positive and gram-negative bacteria such as *Staphylococcus aureus* and *Pseudomonas aeruginosa,* respectively [3,88,89]. Our study found that two immune gene members were down-regulated: *CecA2* and *Drosocin*, antibacterial peptides encoding genes that are expressed during gram-positive and gram-negative bacteria infection [90,91,92]. We suggest that down-regulation of these genes might occur due to the immunity properties of curcumin consumption, which acts as a primary immune defense due to the sole antibacterial properties of this phytochemical [3]. In addition, benzoic acid is associated with vigorous antibacterial activity by inhibiting the proliferation of bacteria such as *Escherichia coli* and *Bacillus subtilis* [93]. In contrast, raffinose-related metabolites inhibit *Streptococcus* [94], and these metabolites were found to be upregulated in our metabolome analysis.

Collectively, these findings enhance the integration between transcripts and metabolomics and provide deeper insights into the molecular mechanisms involved in the response to dietary curcumin consumption.

## 4. Materials and Methods

### 4.1. Drosophila Husbandry and Experimental Diets

Wildtype Berlink-K (8522) genotype *D. melanogaster* (Indiana University, Bloomington, IN, USA) was used for this research. The control diet was prepared using autoclave-sterilized standard cornmeal medium (Nutri-fly Bloomington formulation, Genesee Scientific, San Diego, CA, USA), to which 0.5% propionic acid (*v*/*v*) and 1.5% Tegosept (*w*/*v*) were added as preservatives. To the previously described formulation, a 10 mg·gr^−1^ of curcumin (Sigma-Aldrich C1386, St. Louis, MO, USA) was added for the curcumin diet. All experiments were conducted under controlled conditions with a 12 h light/dark photoperiod at 24 °C. Twenty flies (ten males and ten females) were placed separately in vials with varying diets. Parental flies were removed from the vials after 96 h of egg laying. Larvae were fed until they reached mature adulthood (5 days after eclosion) and then selected for subsequent analysis. Each of the control and curcumin groups was independently replicated five times.

### 4.2. Food Intake and Physiological Assays

Food and physiological analyses were conducted following the methods described [95]. A capillary feeder (CAFE) assay measured the amount of food single flies eat in the control and curcumin diet [96]. Ten flies were placed in standard fly vials with four glass capillaries (53432-706, VWR) filled with control and curcumin diets as described above. Food consumption in the capillaries was measured after 24 h using a caliper. One parallel vial void of flies was used as a control to determine the extent of food evaporation from the capillaries. The total food intake (µL/fly) was calculated as (food consumption − evaporation loss)/number of flies.

The body weight of adult flies was measured in five biological replicates. From each replication, ten male and ten female flies were chosen and weighed individually using an electronic balance (Mettler Toledo #XS64). The weights of the flies were recorded in milligrams (mg), and the mean weight was calculated for each diet. To determine the triglyceride (TG) levels, both groups of adult male and female flies were homogenized in 300 µL of phosphate-buffered saline (PBS-1×) using a high-throughput homogenizer (Qiagen, Hilden, Germany). To quantify TG levels, 20 µL of PBS-1× supernatant was mixed with 200 µL of TG reagent (TR22421 Thermofisher, Waltham, MA, USA), and the resulting mixture was measured at OD 550 nm. Similarly, to measure glucose levels, both adult female and male flies were homogenized in 100 µL of 100 mM PIPES buffer (Sigma P6757, St. Louis, MO, USA) along with porcine kidney trehalose at 5 µL per 2 mL (Sigma T8778). The homogenates were then incubated at 37 °C for 1 h to allow for trehalose to glucose conversion. 10 µL of supernatant was transferred to 100 µL of the Glucose GO assay reagent, and the absorbance was measured at 340 nm using a SpectraMax M2e instrument (Molecular Devices Corp., San Jose, CA, USA). Collected physiological data were analyzed by unpaired, two-tailed *t*-test to detect significant differences among diets and Cohen’s d to understand the effect size by comparing the difference between the two means [97].

### 4.3. Total RNA Extraction and Transcriptomic Analysis

RNA was extracted from the whole-body tissue of control and curcumin-fed flies in triplicate. For each replication, 20 flies (10 females and 10 males) were selected. Before the total RNA was extracted using the Trizol reagent (Thermofisher 15596026), the flies were surface sterilized with 5% sodium hypochlorite. The isolated RNA was assessed for degradation and contamination on 1.2% agarose gels. The quantity of the RNA was measured using the Qubit 3.0 Fluorometer. RNA-Seq libraries were constructed using equal amounts of RNA (1 μg/μL) and the NEBNext Ultra RNA Library Prep Kit for Illumina (New England Biolabs, Ipswich, MA, USA), following the manufacturer’s instructions. The libraries were pair-end sequenced on an Illumina NextSeq 500 system with a 75 bp. Sequences were deposited in the NCBI SRA repository under BioProject ID: PRJNA860149.

Raw reads were quality-checked by using FASTQC and were adapter- and low-quality-filtered (Q value ≤ 30) by using Trimmomatic (version 0.39) [98]. Clean reads were aligned to the *Drosophila melanogaster* genome (http://ftp.flybase.net/releases/FB2020_05/dmel_r6.36/fasta/ (accessed on 15 November 2023)) by using STAR [99]. HT-Seq (https://github.com/htseq/htseq (accesed on 15 November 2023)) was utilized to generate a raw gene count table for annotated genes in the fly genome that were identified with at least one mapped read, counting the reads mapped to each gene feature. For the differential expression analysis, DESeq2 (https://bioconductor.org/packages/release/bioc/html/DESeq2.html (accessed on 15 November 2023)) was employed by comparing control and curcumin diets samples. Threshold values of ≥1 log2FC and a false discovery rate ≤ 0.05 were used to evaluate the differential expressed genes (DEGs).

Gene ontology enrichment (GO) in DEGs was determined using the R package topGO (version 3.17). String (version 2.0.1) and ClueGO (version 2.5.10) were employed to find gene networks in the top 100 up- and down-regulated DEGs [100]. ShinyGO (version 0.77) and the KEGG database were used to identify pathways in DEGs with an FDR cutoff of ≤0.05.

### 4.4. Metabolomics

Metabolomes of flies on control and curcumin diets were carried out in triplicates, with 20 flies (10 females and 10 males) selected for each sample to minimize sex bias. The metabolite profiles were obtained through a gas chromatography–mass spectrometry (GC-MS) system, which included an Agilent 7890 gas chromatograph, an Agilent 5975 MSD, and 7683B autosampler, at the Metabolomics Laboratory of Roy J. Carver Biotechnology Center, University of Illinois at Urbana-Champaign, United States. The AMDIS 2.71 software and a custom-built database comprising 460 unique metabolites were used to evaluate the spectra of all chromatogram peaks. The intensity of the added internal standards during extraction and measured fly body weights was normalized to determine the relative quantification of total fly metabolites.

Metabolites that were undetected in ≥90% of samples in all biological groups were excluded from the analysis. Statistical analyses and data visualization for metabolome data were carried out using MetaboAnalyst 5.0 [101] and the Kyoto Encyclopedia of Genes and Genomes (KEGG). The partial least square (PLS) regression method was employed for the classification of the most important biomarkers in the metabolomics profiling using a discriminant analysis (PLS-DA), and the potential metabolites were screened based on the variable importance in the projection (VIP) scores and selected the compounds greater than 1 (threshold VIP > 1).

### 4.5. Weighted Gene Co-Expression Network Analysis (WCGNA) and Integration of Transcriptome and Metabolome

The co-expression network was constructed using the generated count table from RNA-seq data with the WGCNA package (version 4.2.2). The minModuleSize parameter was set to 30, and the mergeCutHeight was set to 0.25 [102]. Generated hub genes were then subjected to pathway over-representation analysis with DAMs in IMPaLA: Integrated Molecular Pathway Level Analysis (http://impala.molgen.mpg.de (accessed on 30 November 2023)) [38].

## 5. Conclusions

Curcumin is a compound linked to various human health benefits, including antioxidant, anticancer, and neuroprotective properties. Our research has shown that curcumin affects several bodily functions, such as redox homeostasis, lipid and carbohydrate metabolism, perception and gustatory systems, the immune system, fecundity, and mating behavior at both the gene expression and metabolite accumulation levels. However, we need to explore further and understand the effects of chronic curcumin administration and how different dosages can be beneficial or detrimental to support our results. Moreover, we need to evaluate potential sex-specific influences on these processes, allowing us to understand how curcumin might affect males and females differently, impacting processes such as chronic disease development, dietary preferences, and reproduction. Additionally, research directions on curcumin’s secondary benefits may help us validate its demonstrated antimicrobial properties.

## Figures and Tables

**Table 1:** Selected genes with differential expression in response to curcumin diet and their biological process.

Gene ID	Gene Symbol	Differential Expression	Function	Biological Process
FBgn0004552	*Akh*	Up	Hormone activity	Glucose and lipid homeostasis
FBgn0038449	*CG17562*	Up	alcohol-forming very long-chain fatty acyl-CoA reductase activity	long-chain fatty-acyl-CoA metabolic process
FBgn0033216	*CG1946*	Up	diacylglycerol O-acyltransferase activity	Triglyceride biosynthetic process
FBgn0033204	*CG2065*	Up	NADP-retinol dehydrogenase activity	Retinal metabolic process
FBgn0038469	*CG4009*	Up	Peroxidase activity	Response to oxidative stress
FBgn0034909	*CG4797*	Up	Transport activity	Carbohydrate transport
FBgn0034142	*CG8306*	Up	alcohol-forming very long-chain fatty acyl-CoA reductase activity	long-chain fatty-acyl-CoA metabolic process
FBgn0034756	*Cyp6d2*	Up	Oxidoreductase activity	Stress response
FBgn0031461	*dawdl*	Up	Cytokine activity	cellular response to nutrient levels
FBgn0029172	*Fad2*	Up	Oxidoreductase activity	Lipid metabolic process
FBgn0283427	*FASN1*	Up	fatty acid synthase activity	Lipid metabolic process
FBgn0039114	*Lsd-1*	Up		Lipid storage
FBgn0023479	*tequila*	Up	serine-type endopeptidase activity	Glucose homeostasis
FBgn0030748	*Traf-like*	Up	Protein binding activity	positive regulation of lipophagy
FBgn0038069	*CG11608*	Down	Triglyceride lipase activity	Lipid metabolic process
FBgn0042207	*CG18530*	Down	Triglyceride lipase activity	Lipid metabolic process
FBgn0038070	*CG6753*	Down	Triglyceride lipase activity	Lipid metabolic process

**Table 2:** The top 10 integrated pathways between transcriptomic and metabolomic of *D. melanogaster* in response to curcumin diet.

Biological Pathway	Function	Genes	Gene Symbol	Metabolites	Metabolite Name	Joint (*p*-Value)
Arginine Proline metabolism	Biosynthesis of arginine, ornithine, proline, citrulline and glutamate	5	GCLC; GAD1; ALDH7A1; OAT; ODC1	9	Glycine; a-Ketoglutaric acid; UREA; Succinic acid; Aspartic acid; Spermidine; GABA; Putrescine; Fumaric acid	4.26 × 10^−11^
Alanine and aspartate metabolism	Source of energy, gluconeogenesis, and NH3 transport	1	GAD1	7	Tricarballylic acid citric; pyruvic acid; a-Ketoglutaric acid; Aspartic acid; Succinic acid; Asparagine; Fumaric acid	3.87 × 10^−8^
Glutamate Glutamine metabolism	Substrate for nucleotide synthesis, NADPH, antioxidants, and NH3 transport	3	GCLC; GAD1; ALDH7A1	6	pyruvic acid; Glycine; a-Ketoglutaric acid; Succinic acid; Aspartic acid; GABA	3.91 × 10^−8^
G Protein Coupled Receptors downstream signaling	Activate cAMP and inositol phospholipids signaling	1	MMP1	17	Nicotinic acid; glycerol-3-p; glycerol; Histamine; Palmitic acid; Pentadecanoic acid; Dopamine; stearic acid; a-Ketoglutaric acid; GABA; Tetradecanoic acid; Dodecanoic ACID; 9-Hexadecenoic acid; Succinic acid; 9-Octadecenoic acid; Adenosine; sucrose	8.02 × 10^−8^
Protein digestion and absorption	Proteolytic activity for amino acids and small peptides production	1	COL4A1	10	Histamine; Threonine; Glycine; Asparagine; valine; isoleucine; Aspartic acid; Tyrosine; Putrescine; Histidine	8.04 × 10^−8^
Glycine_serine and threonine metabolism	Essential amino acid and intermediate of glycolysis	3	GNMT; SRR; ALDH7A1	7	homoserine; Threonine; pyruvic acid; Glyceric acid; Glycine; glyoxylic acid; Aspartic acid	1.32 × 10^−7^
Signaling by GPCR	Promote heterotrimeric GTP-binding protein activation, acts as a guanine nucleotide exchange factor	1	MMP1	18	NICOTINIC ACID; glycerol-3-p; glycerol; Histamine; Palmitic acid; Pentadecanoic acid; Dopamine; stearic acid; Dodecanoic ACID; GABA; Tetradecanoic acid; a-Ketoglutaric acid; 9-Hexadecenoic acid; Succinic acid; 9-Octadecenoic acid; Adenosine; KYNURENIC ACID; sucrose	3.74 × 10^−7^
Glyoxylate and dicarboxylate metabolism	Metabolism of fatty acids and glutamate	1	CAT	10	Tricarballylic acid#citric acid; Glycolic acid; pyruvic acid; Glyceric acid; Glycine; a-Ketoglutaric acid; glyoxylic acid; oxalic acid; Succinic acid; Phosphoglycolic acid	3.80 × 10^−7^
Purine Metabolism	Maintain optimal level of nucleotides	2	GART; PFAS	9	Hypoxanthine; Xanthine; Adenine; Guanosine; Glycine; Aspartic acid; Inosine; Adenosine; Fumaric acid	7.91 × 10^−7^
Purine Nucleoside Phosphorylase Deficiency	Immune system	2	GART; PFAS	9	Hypoxanthine; Xanthine; Adenine; Guanosine; Glycine; Aspartic acid; Inosine; Adenosine; Fumaric acid	7.91 × 10^−7^

## Data Availability

The RNA sequencing data generated in this study are available in the NCBI database under the BioProject accession number PRJNA1106560.

## Supplementary Materials

The following supporting information can be downloaded at: https://www.mdpi.com/article/10.3390/ijms25126559/s1.
