# Supplementary material for: Dietary Curcumin Intake and Its Effects on the Transcriptome and Metabolome of Drosophila melanogaster
Source: Int J Mol Sci. 2024 Jun 14;25(12):6559. doi: 10.3390/ijms25126559 (PMC11203963; doi:10.3390/ijms25126559)
Supplement: Supplementary file 1 [file ijms-25-06559-s001.zip › Supplementary Figures.pdf]

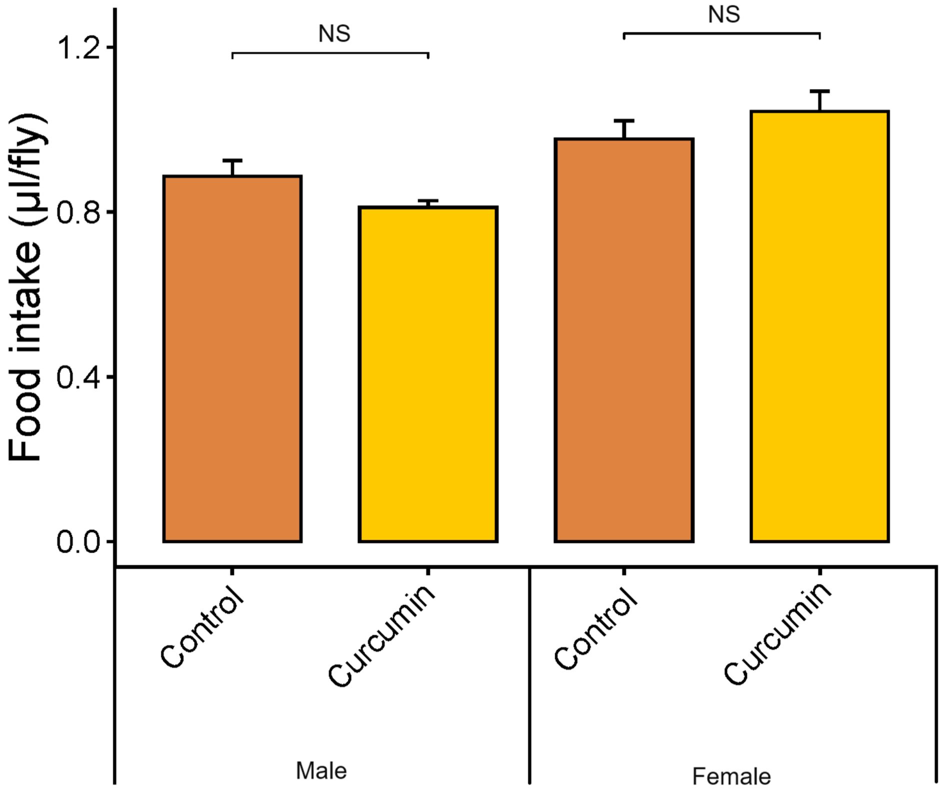

Figure S1. Male and Female food intake of *D. melanogaster* on control and curcumin diets. CAFE assay in (µL/fly). Data are mean ± SEM for n = 5 independent replicates. *, $p \leq 0.05$, **, $p \leq 0.01$, $p \leq 0.001$, NS, not significant, compared to control versus treatment.

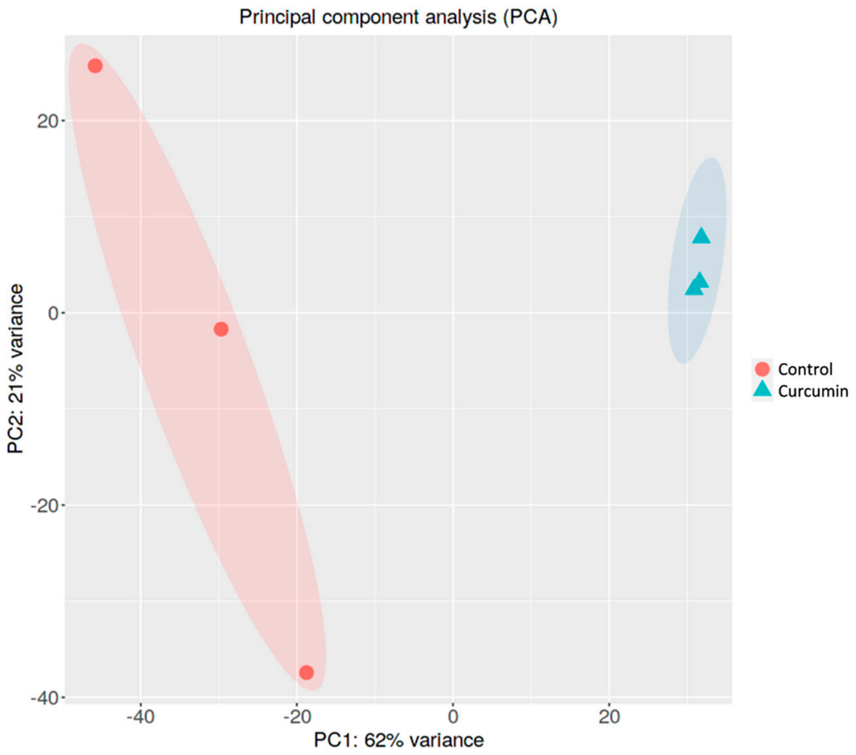

Figure S2. The 2-D principal component analysis score plot demonstrates the statistical clustering of genes in response to a curcumin (blue) and control (red) diet.

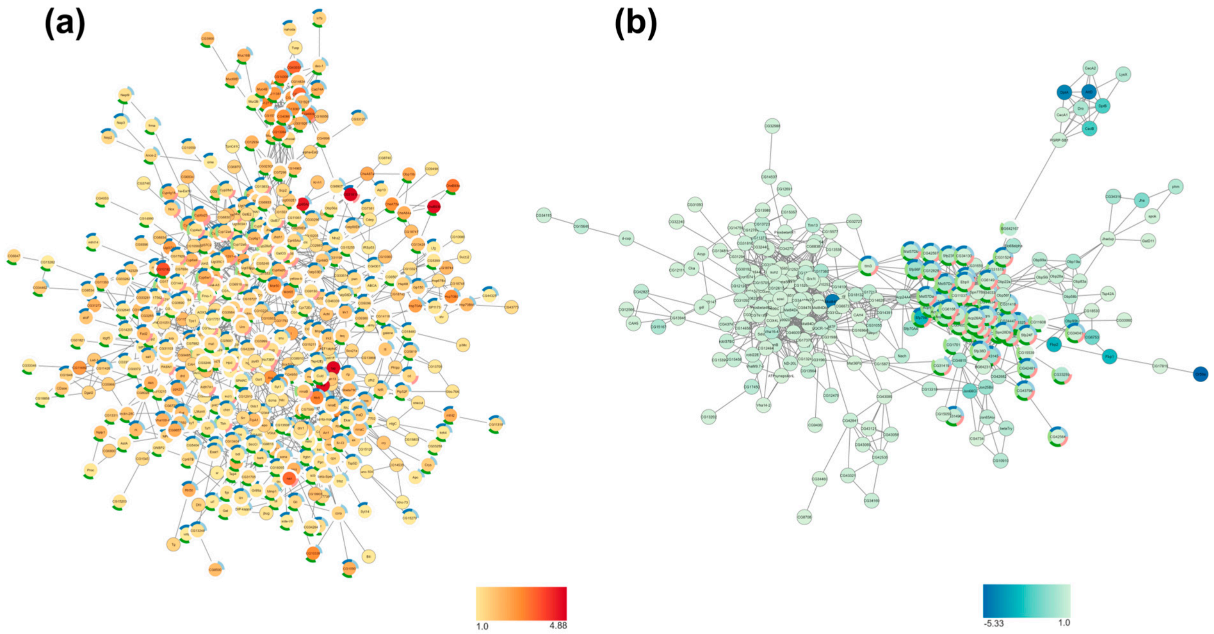

Figure S3. Gene links of the top 100 (a) up- and (b) down-regulated genes in response to curcumin treatment with a cutoff of $p < 0.05$.

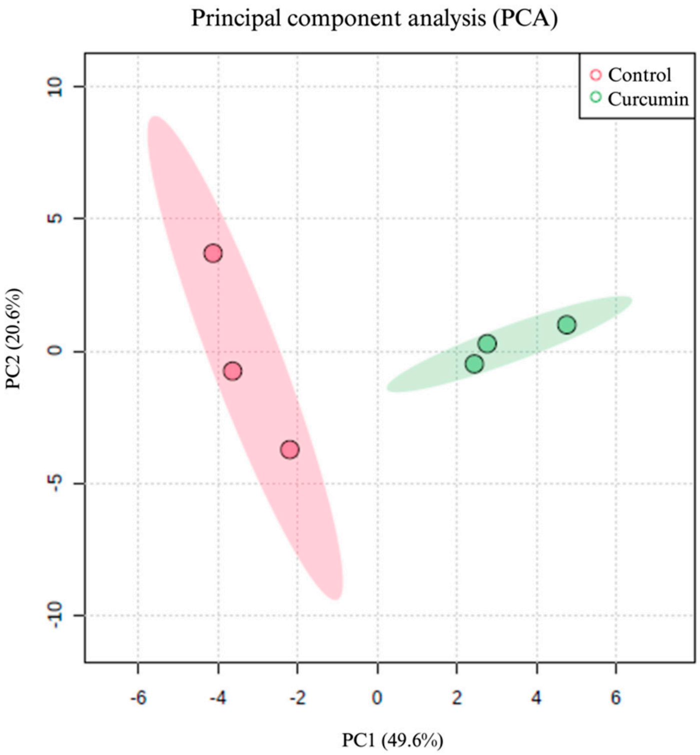

Figure S4. Principal component analysis (PCA) on the metabolomics profiles of Drosophila under a regular diet and a curcumin-supplemented diet.
